# Characterization of Root Hair Curling and Nodule Development in Soybean–Rhizobia Symbiosis

**DOI:** 10.3390/s24175726

**Published:** 2024-09-03

**Authors:** Wei Lu, Xiaochan Wang, Weidong Jia

**Affiliations:** 1 Department of Agricultural Engineering College, Jiangsu University, Zhenjiang 212013, China; 2 Department of Engineering College, Nanjing Agricultural University, Nanjing 210031, China; wangxiaochan@njau.edu.cn

**Keywords:** soybean, rhizobia, microrhizotron, root hair, in situ

## Abstract

Soybean plants form symbiotic nitrogen-fixing nodules with specific rhizobia bacteria. The root hair is the initial infection site for the symbiotic process before the nodules. Since roots and nodules grow in soil and are hard to perceive, little knowledge is available on the process of soybean root hair deformation and nodule development over time. In this study, adaptive microrhizotrons were used to observe root hairs and to investigate detailed root hair deformation and nodule formation subjected to different rhizobia densities. The result showed that the root hair curling angle increased with the increase of rhizobia density. The largest curling angle reached 268° on the 8th day after inoculation. Root hairs were not always straight, even in the uninfected group with a relatively small angle (<45°). The nodule is an organ developed after root hair curling. It was inoculated from curling root hairs and swelled in the root axis on the 15th day after inoculation, with the color changing from light (15th day) to a little dark brown (35th day). There was an error between observing the diameter and the real diameter; thus, a diameter over 1 mm was converted to the real diameter according to the relationship between the perceived diameter and the real diameter. The diameter of the nodule reached 5 mm on the 45th day. Nodule number and curling number were strongly related to rhizobia density with a correlation coefficient of determination of 0.92 and 0.93, respectively. Thus, root hair curling development could be quantified, and nodule number could be estimated through derived formulation.

## 1. Introduction

Soybeans provide abundant protein and oil for human and animal diets. During soybean growth, nitrogen plays a critical role and is demanded in large amounts [1,2]. Soybeans can form symbiotic associations with rhizobia as nodules to fix atmospheric N_2_ into ammonia [3,4]. Efficient plant root nodulation and subsequent N_2_ fixation provide a large proportion of nitrogen for soybean plant development [5]. Currently, the amount of symbiotically fixed N_2_ is about 50–60% of that demanded in the soybeans’ lifetime [6]. This natural process in rhizobia–legume symbiosis is of vital importance in reducing the application of chemical fertilizer and providing a clear nitrogen source for soybean development.

The root hair is the initial infection site for soybean (legume plants)–rhizobia symbiosis [7,8,9]. During nodule formation, rhizobia bacteria always attach to root hairs and induce root hair deformation [10,11]. Bacteria form bacteroids and symbioses and ultimately form nodule organs with the ability to fix N_2_ [12,13]. The inoculation of rhizobia also affects root morphology and structure [14,15,16,17]. Some research [14] found that soybean rhizobia increased the size and number of cortical cells in the root meristem and elongation areas, which expanded root hair density, expanded root hair area, and produced more nodules. The root hairs are deformed in many shapes, such as curling, wiggling, branching, and shepherding, which could entrap bacteria [18,19,20,21]. Soybean root hair deformation responded to the increase of rhizobia (nod factor) concentration [22], but whether more deformed root hairs lead to more nodulation has not been elucidated by the literature. In addition, little knowledge is available in the process of soybean root hair deformation, nodule initial, and nodule development over time in situ.

Therefore, it is very important to study root hair deformation progress in soybean–rhizobia symbiosis, which will help to understand the mechanism of root hair deformation. Root hairs are a very tiny and sensitive part of rhizobia–legume symbiosis [23,24]. The length of root hairs ranges from tens of microns to hundreds of microns, but they are only dozens of microns in width. Owing to the difficulty in observing roots in the soil, most research was carried out with a destructive method or in an agar/solution environment observed using light microscopy [17,25,26]. It either had the limits of losing root hairs during sampling or could not represent real growth under a soil environment. In addition, these methods did not support successive studies on the same roots over time. X-ray computed tomography (X-CT) [27], nuclear magnetic resonance (NMR) [28], and electrical impedance tomography (EIT) [29] were not intended for such tiny root hair traits. To make it applicable to focus on detailed roots such as root hairs, rhizotrons, and minirhizotrons, methods were improved and adapted to a smaller scale by amplifying the local area [30,31,32,33].

In this study, we intended (1) to observe root hair traits and root nodulation development under different rhizobia densities in rhizobia–legume symbiosis over time, with the previously designed microrhizotron (1.5 cm^3^ in volume) in situ, (2) to document curling of root hairs after rhizobia inoculation, (3) to analyze the relationship between root hair curling/ root nodule and rhizobia density, and thus comprehend the mechanism of root hair deformation and nodulation.

## 2. Material and Methods

### 2.1. Plant Materials and Root Hairs In Situ Observation

**Figure 1:**
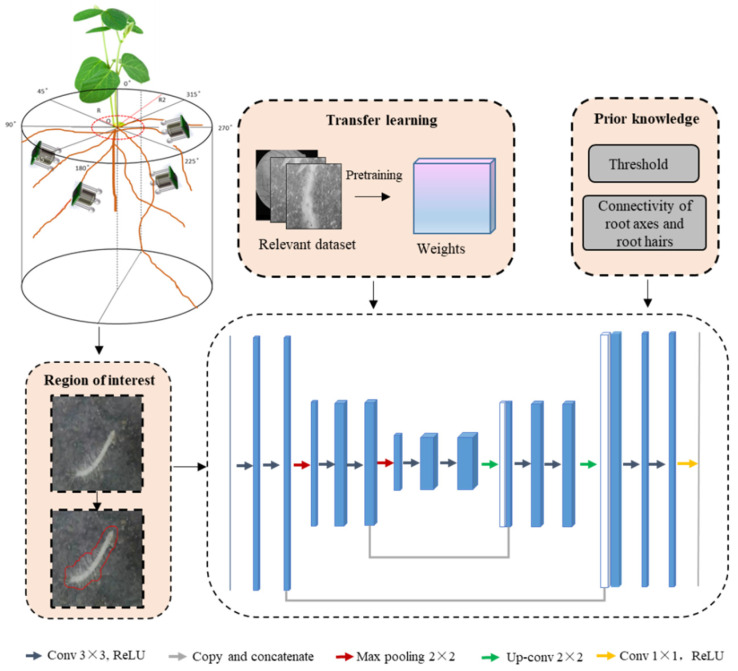
Root image capture and image processing with the proposed deep learning model based on prior knowledge and region of interest.

Soybean (*Glycine max cv. Williams* 82) seeds were surface sterilized by treating with 7% NaClO for 5 min followed by 70% ethanol for 4 min. The seeds were then rinsed three times with sterile deionized water. After sterilization, the seeds were planted in a greenhouse at Nanjing Agricultural University, Engineering College (32°18′ N, 118°46′ E) in 5 experimental zones filled with soil taken from the local crop growing area (brown clay soil, organic matter: 318 g/1000 g, total N: 1.6 g/1000 g). The soil used was filtered 3 times with a 1 cm opening filter and then was sterilized at 80 °C for defaunation. One-week-old seedlings were installed with microrhizotrons (1.1 cm × 1.1 cm × 1.2 cm). Microrhizotrons are a kind of tiny root observation and processing system designed and evaluated for non-destructive root observation, composed of a micro-camera, optical amplifier, lighting, and a circuit board [31]. The size of the microrhizotron is 1.1 cm × 1.1 cm × 1.2 cm, which can be preset at multi-points and observe root growth for a long time. Effective installation to intercept with more roots was carried out according to the soybean lateral root initiating regulations: the roots of soybean belong to tetrarch, where lateral roots initiate from the pole pericycle cells opposite the protoxylem, and generally, lateral roots are spaced along the longitudinal axis in four lines. Details are also in [32,34]. So, four microrhizotrons were preset around each plant root at a depth of 50 mm and 100 mm, and at each depth, two microrhizotrons were placed along the direction of the cotyledons. Soil compaction was tamped to 0.80–0.83 kg/cm^2^ after installation (Figure 1).

*B. japonicum strains Parasponia Bradyrhizobium* strain ANU289 (Institute of Plant Protection, Jiangsu Academy of Agricultural Sciences) at very low (10^5^–10^6^ viable cells per Leonard jar), low (10^6^–10^7^ viable cells per Leonard jar), medium (10^7^–10^8^ viable cells per Leonard jar), and high (10^8^–10^9^ viable cells per Leonard jar) inoculant doses were used in this experiment. Viable cell numbers were determined by dilution platings on a normal Rhizobium growth medium. Two-week-old seedlings were flood-inoculated with water (control), very low, low, medium, and high ANU289 suspension (200 mL/plant), named Control group, A group, B group, C group, and D group. Monitoring was carried out every day from inoculation to nodule formation (50th day after inoculation). The elongating zone and mature zone with growing root hairs were the target areas for observation. The captured images each day (16:00–17:00) were transmitted wirelessly to the terminal device for further processing. Root hair images were segmented based on deep learning and prior knowledge (Figure 1) (Supplementary File S1); see also in [33]. The proposed model was implemented in Python 3.6, where Keras 2.2.4 and TensorFlow-GPU 1.15.0 were used. The hardware platform is the Intel(R)Core(TM)i7-7700 CPU@3.60 GHz, 8.00 GB memory. GPU is NVIDIA RTX3060, 12 GB memory.

### 2.2. Root Image Processing (Root Hair and Nodule) and Root Hair Separation

**Figure 2:**
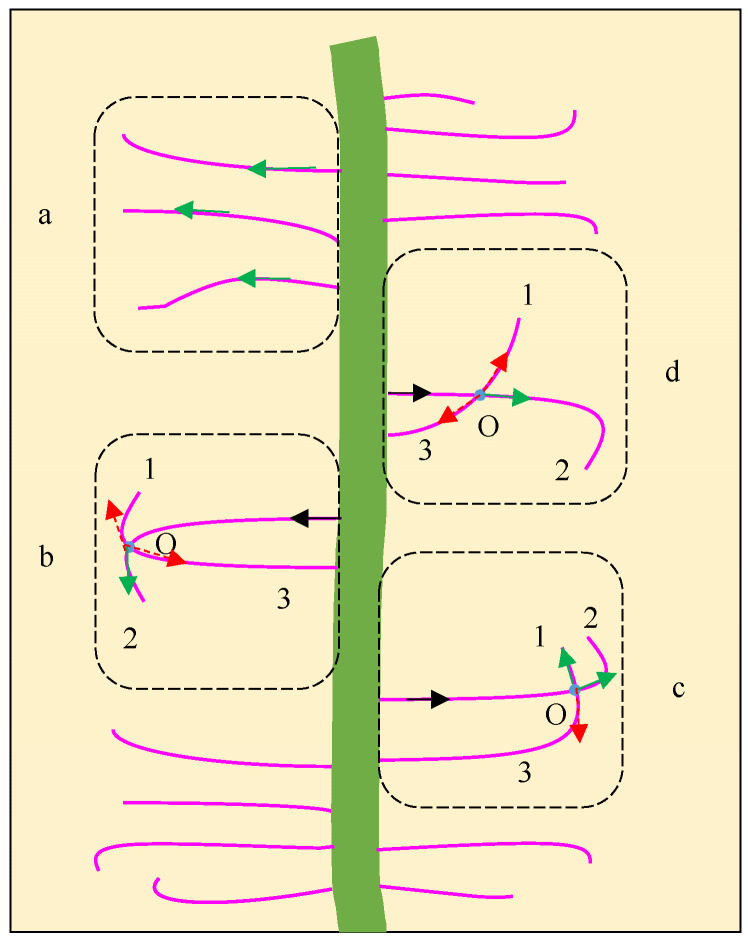
Crossing and overlapping root hair separation. a, b, c, and d are different crossing or overlapping types for root hairs. The arrow in green is the correct path, and the arrow in red is the wrong path for root hair searching.

After segmentation, root hairs were thinned to a single pixel using the Zhang–Suen thinning algorithm [35]. There were some small gaps in the root hair, and they were connected by approximating the nearest neighbor root hair with a similar slope [36,37]. In order to calculate the root curling angle, root hairs should be separated from one another. There were several patterns for root hair curling and crossing, as shown in Figure 2. The crossing and overlapping root hairs were separated based on the following rules: root hair connectivity and continuity, and plant roots and curling growing continuously with small differences in root angle [38,39].

As shown in pattern a in Figure 2, no crossing or overlapping occurs, and there is only one path for the search from the beginning to the end. In pattern b, there is more than one path for the search, while only path 2 is the correct one. Both path 1 and path 3 are certainly wrong because the angle difference changes dramatically with a sudden break point. In c, since the crossing and overlapping are at the end, the threshold was defined as 80°. If the angle is less than 80°, it is regarded as root hair; otherwise, it is not regarded as a true path. However, in pattern d, it is a little hard to distinguish path 1 from the correct one since it has a moderate slope and might be mixed with real root hair curling. It was found during the experiment that the root hair curling always occurred at the root hair end; thus, if the crossing point is within 1/2 of the root, the threshold should be set smaller (30°). In detail, the root hair separation algorithm is presented in the following steps:

Step 1: Root hairs thinning

Root hairs were thinned and separated from root axes using the Zhang–Suen thinning algorithm and our proposed pruning method [33]. The boundary coordinate of an axis was stored for the root hair connecting with the root axis.

Step 2: Root hair connecting with root axis

After thinning, root hairs were shortened, and the root axis was thinned. Given the rule that a root hair could only be connected to one axis, gaps between root hairs and the root axis were connected by approximating the nearest neighbor, considering the continuously growing features of root hairs. The boundary coordinate of an axis was compared with the endpoint of a root hair (single pixel) to ensure they were neighboring.

Step 3: Searching and separating

The search for the root hair started from the root axis (boundary coordinate) along the single-pixel root hair (black arrows). When there was more than one path for the search forward, the angle difference was calculated and compared. All the root hairs should follow the principle that roots grow continuously with no sudden break point. If the crossing point is within 1/2 of the root, the angle difference threshold was set as 30°. If it is at the end, where curling appears occasionally, the threshold was set as 80°. Root hairs were separated from one another for further calculation of the root hair curling angle (Supplementary File S2).

### 2.3. Root Hair Curling Angle Calculation

**Figure 3:**
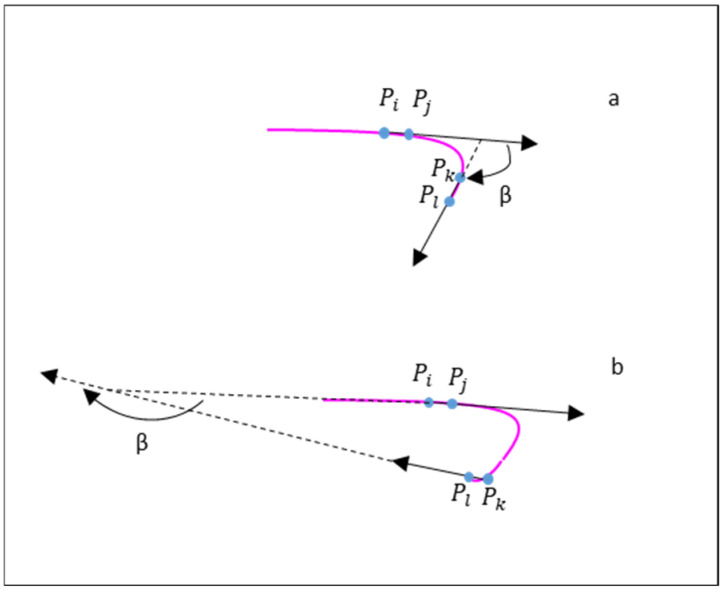
Root hair curling angle calculation. (**a**,**b**) are two patterns of root hair curling.

When the root hair had a small or moderate curling angle (Figure 3a), the angle change was relatively small or moderate. When the root hair had a large curling angle (Figure 3b), the changed direction curling angle could reach more than 225° (Supplementary File S2). The root hair curling angle represents the changed angle from the vector $P_{i}P_{j}$ and $P_{k}P_{l}$ (Figure 3). It is defined as:

(1) $$\beta \;=\arccos\;P_{i}P_{j}\cdot P_{l}/(|P_{i}P_{j}|*|P_{k}P_{l}|)$$

where *β* is the angle between the vector $P_{i}P_{j}$ and $P_{k}P_{l}$, · is the inner product, and *d*L is root hair length from point $P_{i}$ to $P_{k}$.$\;P_{i}$ is selected as the 1/2 root hair here since root hair curling always occurs at the root hair end in soybean–rhizobia symbiosis. $P_{j}$ is the point 5 pixels forward, $P_{l}$ is the endpoint, and $P_{k}$ is the point 5 pixels backward.

### 2.4. Observed Nodule and Real Nodule Conversion

**Figure 4:**
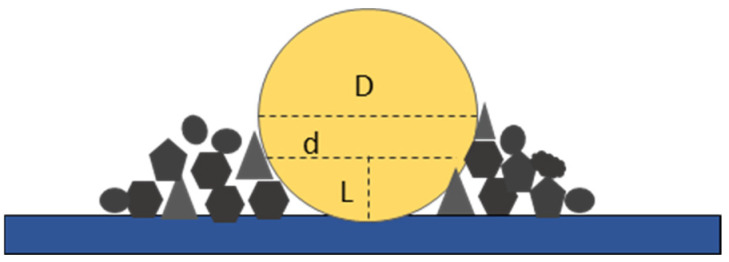
Observed nodule and real nodule. *D* is the true diameter of nodulation, *d* is the diameter perceived by the camera or human eye, and *L* is the visible depth of the soil.

Root length and diameter were calculated through pixel counting and calculation automatically on segmented images [31]. When the diameter of the root nodule is larger than the visual soil depth (*L*), the diameter perceived by observers (*d*) will be smaller than the real size of the nodule (*D*), as shown in Figure 4. Accurately, the minirhizotron and microrhizotron could only see into the soil about 1 mm [38], while the diameter of the root nodules could reach more than 4 mm. Therefore, the real root nodules are defined as:

(2) $$D=\frac{{(4L}^{2}+d^{2})}{4L}$$

where *D* is the real diameter of nodulation, *d* is the diameter perceived by the camera or human eye, and *L* is the visible depth of the soil.

## 3. Result

### 3.1. Root Hair Curling Angle in Different Rhizobia Density

**Figure 5:**
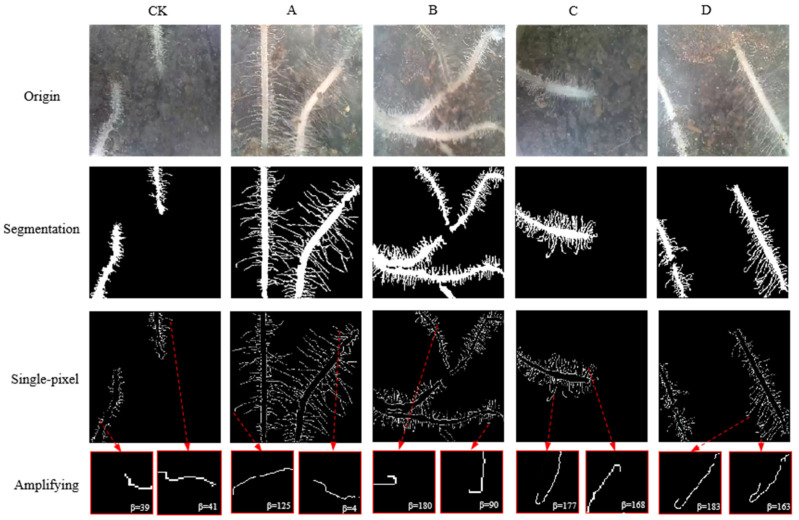
Root hair curling angle in different rhizobia densities (8th day). Amplifying is amplified root hairs in a single-pixel image. The arrow points to curling root hairs and its amplified image.

The root hair was deformed on the 5th day after inoculation for several days. The root hair curling angle under different rhizobia densities (8th day) is shown in Figure 5. It could be observed that most root hairs were tender containing a lot of water, which made the roots seem semitransparent. Visually, root hairs were not all straight. Root hairs in groups A and B showed some deformed root hairs, and group C had the most curling root hairs. But when higher rhizobia were applied in group D, its root hair curling was not more than that in group C, which indicated that a higher density of rhizobia than group C did not stimulate more root hair curling. There were several types of root hair deformation in the soybean–rhizobia symbiosis, such as wiggling, waving, and branching root hairs. The root hair curling angle was calculated and studied in detail. The root hair curling angle could reach more than 225°, but a large root hair curling angle has never been seen in the control group.

**Figure 6:**
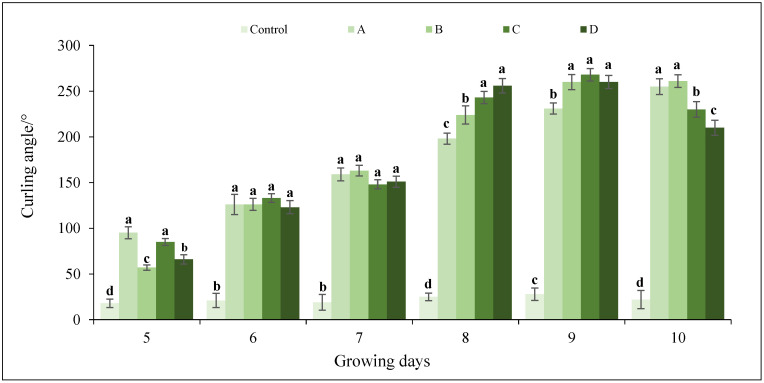
Root hair curling in infected and non-infected groups. Days refer to inoculated time. Bars indicate standard deviations (N = 30 root hairs from 5 different plants). Different letters indicate significant differences by the Student–Newman–Kuels test (*p* < 0.01).

The dynamic root hair curling angle with the increase of rhizobia density and root growing stage is illustrated in Figure 6. Root hair curled on the 5th day after inoculation and reached the highest curling angle on the 8th day. The largest curling angle was about 268°, appearing on the 9th day in group C. For groups A and B, root hairs further curled more or less on the 10th day, but the degree was very small. The higher density of rhizobia did not stimulate larger root hair curling angles in group D.

### 3.2. Root Hair Curling Angle over Time

**Figure 7:**
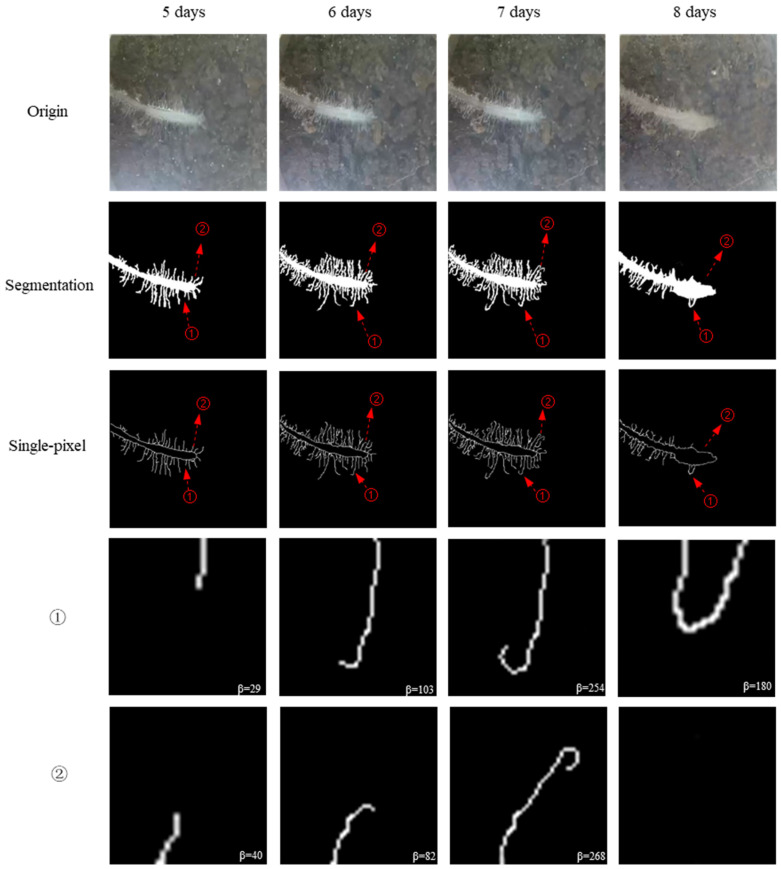
Root hair curling angle over time. ① and ② are amplified root hairs in a single-pixel image. Days refer to inoculated time.

Soybean root hairs deformed from the 5th day to the 8th day are shown in Figure 7 (taking group C as an example). At early inoculation (5th day and 6th day), root hairs were short and almost straight. Then, they elongated and turned a little bit, bending from the middle to the end. After a period of inoculation (7th day), root hairs bent severely. Some root hairs became waved, and some became hooked or curled at the end, with a relatively large angle of more than 180°. On the 8th day, a little swelling could be observed. In ①, the curled root hair could not be seen obstructed by the swelling part. In ②, the full tight curl of root hair could be partly observed.

**Figure 8:**
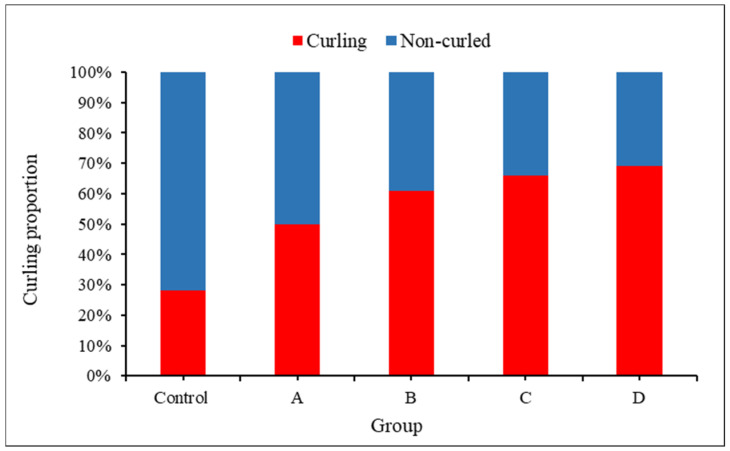
Root hair curling proportion on the 8th day after inoculation (N = 30 root hairs from 5 different plants).

The curling angle in the infected group was larger than that in the uninfected group on the 6th day (Figure 6 and Figure 7). In the uninfected group, the curling angle did not change much over the range of the growing stage. However, it was worth noting that even in the uninfected group, some small deformations occurred, which implied that root hairs were not always straight and a threshold should be set to determine curled root hairs in soybean–rhizobia symbiosis. Figure 8 displays the root hair curling proportion on the 8th day. There was a proportion of curling root hairs in the control group. This was because not all the root hairs were straight, and deformation with a small curling angle was also regarded as curling root hairs. So, in further analyses, only a curling angle over 90° was taken into consideration as real curling root hairs.

### 3.3. Nodule Development over Time and Diameter Calibration

**Figure 9:**
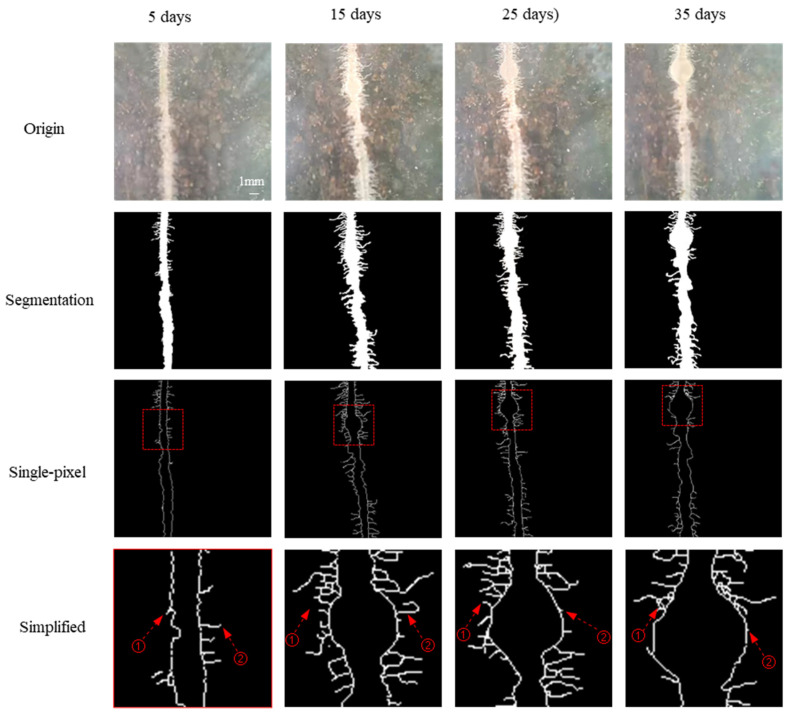
Nodule formation in soybean–rhizobia symbiosis over time (C group). ① and ② are amplified root hairs on the nodule. The red box points to the swelling of roots and curling root hairs.

A nodule formed after root hair curling, as displayed in Figure 9. At early inoculation on the 5th day, root hairs were almost straight. Root hair ② turned into a tight curl on the 15th day. And on the 25th day and 35th day, root hair ② could not be observed. Not all the root hairs turned curly during the inoculation (①, for example). The nodule is an organ developed after inoculation and root hair curling. The swelling was observed on the 15th day, and it was initiated from the root axis with a light color similar to that of the root hair and root axis. The swelling part grew large, and the nodule turned a little dark on the 25th day. On the 35th day, the root nodule became large, and root hairs on the swelling part could not be seen.

The nodule volume developed fast during the first four stages and reached 3 mm in diameter on the 35th day. On the 45th day, the nodule was about 5 mm. When the root nodule diameter was larger than the visual soil depth (1 mm), the diameter perceived by observers was less than the true size of the nodule. As the nodule volume grew, the difference between the observed value and the calculated value became large. Compared with the digging method (Table 1), the calculated errors were 3.94%, 4.84%, 2.08%, 6.16%, and 7.45% on the 20th day, 25th day, 30th day, 35th day, 40th day, and 45th day, respectively, which were much smaller than the observed errors 10.24%, 6.05%, 13.51%, 12.79%, and 14.58%. Thus, the errors could be reduced by converting the observed diameter to the real diameter when the diameter of the root nodule reached more than 1 mm.

### 3.4. Relationship between Nodule Number/Diameter and Rhizobia Density

**Figure 10:**
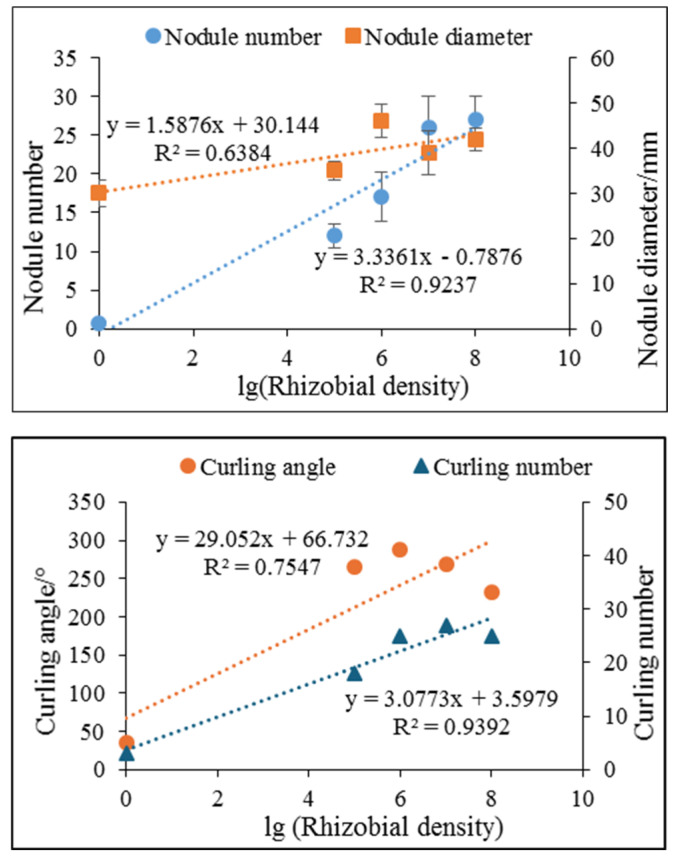
Relationship between nodule number per plant/diameter and rhizobia density (N = 30 nodules/root hairs from 5 different plants in each density).

Linear regression analysis was conducted between the nodule/curling hairs and the rhizobia density applied in the soil (Figure 10). Regressions for nodule number per plant versus rhizobia density had a high correlation coefficient of determination (R^2^ 0.92), suggesting that nodule number was strongly related to rhizobia density. Regressions for curling number versus rhizobia density also had a relatively high correlation coefficient of determination (R^2^ 0.93), suggesting that curling number was strongly related to rhizobia density. On the contrary, the coefficient of determination for nodule diameter versus rhizobia density was 0.63, and the coefficient of determination for curling angle versus rhizobia density was 0.75. These results demonstrated that high or low rhizobia density did not affect nodule diameter or root hair curling angle a lot, but it did determine curling number and nodule number. Thus, the experimentally derived equation quantitatively described nodule number as a function of rhizobia density. For non-destructive and long-period experiments, nodule numbers could be estimated through the linear relationship.

## 4. Discussion

### 4.1. Principal Findings and Comparison with Other Studies

This study focused on root development progress in soybean–rhizobia symbiosis in situ. As far as we are concerned, this is the first accessible literature about in situ root nodule forming subjected to different rhizobia densities in soybean–rhizobia symbiosis. From the results of the experiments, it was evident that rhizobia density affected soybean root hair deformation and nodule formation. Consistent with many research studies [11,22], root hair curling extent increased with the increase of rhizobia density. However, in this research, it was found that nodule number and curling number were strongly linear in relation to rhizobia density. There were several types of root hair deformation in soybean–rhizobia symbiosis, including swelling, wiggling, bulging, curling, and branching root hairs in many studies [20,22]. Compared with these studies, in addition to the qualitative description, this study quantitatively elucidated root hair curling angles in a soil environment non-destructively.

It was interesting that even root hairs in the uninfected group were not straight, with a relatively small curling angle (within 45°), while in the infected group, the largest root hair angle reached 268°. Our result was similar to Roy’s [40] conclusion that not every root hair was straight, with evidence of genetic mutants and root hair stress phenotypes. However, due to technological bottlenecks that inhibit the discovery of root hairs, further studies have not been carried out to prove it. To some extent, our findings about non-straight root hairs in the uninfected group gave some evidence to these hypotheses. It was worth noting that when the nodule finally formed in the late stage (45th day), root hairs were rare and could not even be seen.

The nodule is the ultimately formed organ in soybean–rhizobia symbiosis with the ability to fix N_2_. Throughout our observations in this research, the nodule began to swell on the 15th day and reached 5 mm in diameter on the 45th day, with its color changing from light to a little dark. The field of view into soil has been a problem for large diameters in all the minirhizotrons. Usually, it was regarded as 2–3 mm in depth, but some research reported that for accurate data, the depth of view into the soil should be much smaller [38]. Considering the difference between visual soil depth and perceived diameter, a nodule diameter larger than 1 mm was converted to a real diameter with the formulation. The deviation was reduced, and a more accurate nodule diameter was acquired through the conversion formulation. After conversion, the largest nodule could reach 5 mm in diameter. Further, the relationship between nodule number, nodule diameter, and rhizobia density indicated that nodule number was strongly related to rhizobia density. For a long period, the experiment that concerns nodule numbers could be estimated through the linear relationship instead of digging up and destroying the plant. Thereby, the nodule diameter could be observed and converted to the real diameter.

### 4.2. Strengths and Limitations

This study aimed at characterizing root hair curling and nodule development subjected to different rhizobia densities in soybean–rhizobia symbiosis. One of the strengths was that it enabled local and dynamic change of root hair deformation during the soybean–rhizobia symbiosis nodule process. Nodule development, including diameter and color, could be observed from swelling to noduling for a long period. Compared with digging or plants growing in liquid and observing with microscopy [17,22,41], plants growing in soil conditions could represent a real response to rhizobia. Furthermore, this study not only paid attention to the shapes of root hairs but also quantitatively described each root hair curling angle throughout the growing stages. At last, the solution to acquire nodule number non-destructively was provided through a linear relationship, and nodule diameter was made more accurate with the conversion formulation.

This study has some limitations. Seedlings were inoculated with the same strain of ANU289. Different strains should be applied to test and verify the promotion and reduction of nodulation in future studies.

### 4.3. Implications and Potential Application

The nodule is the most important outcome of soybean–rhizobia symbiosis. Observing the nodule development process and calculating the nodule number according to the linear relationship were enabled in this study, which could be applied to evaluate nodule ability in soybean–rhizobia symbiosis. Compared with digging and root hair deformations observed using light microscopy, this method is time-saving and easy to carry out when high throughput is needed during soybean–rhizobia symbiosis breeding and screening. The proposed method could be used multi-purposely, where local detailed root hair deformation is expected.

## 5. Conclusions

This study depicted root hair and nodule development progress subject to different rhizobia densities in soybean–rhizobia symbiosis in a soil-based environment. Induced rhizobia caused root hair curling, and the deformation reacted in a time series, including root hair elongation, a small amount of bending, and severe root hair bending. The curling root hairs turned thick and even swelled before nodule formation. The nodule began to swell on the 15th day and reached 5 mm in diameter on the 45th day, with its color changing from light to a little dark brown. Nodule number was strongly related to rhizobia density. This relationship is time-saving and easy to acquire nodule diameter and estimate nodule number without destroying the plant, especially when a high throughput and successive experiment is needed during the screening of soybean–rhizobia genotypes with high nitrogen-fixing ability.

## Figures and Tables

**Table 1:** Relative error of observed diameter and calculated diameter with measured diameter (N = 30 nodules from 5 different plants). Different letters indicate significant differences by the Student–Newman–Kuels test (*p* < 0.01).

			Days after Inoculation			
	20	25	30	35	40	45
Observed diameter (mm)	0.71 ± 0.03 a	1.14 ± 0.06 b	2.33 ± 0.11 b	3.33 ± 0.07 b	3.82 ± 0.12 c	4.16 ± 0.15 b
Calculated diameter (mm)	⁄	1.32 ± 0.09 a	2.36 ± 0.06 b	3.77 ± 0.09 a	4.65 ± 0.7 a	5.33 ± 0.13 a
Measured diameter (mm)	0.68 ± 0.02 a	1.20 ± 0.03 b	2.61 ± 0.08 a	3.85 ± 0.08 a	4.08 ± 0.13 b	4.37 ± 0.13 b
Relative error vs. observed	⁄	10.24%	6.05%	13.51%	12.79%	14.58%
Relative error vs. calculated	⁄	3.94%	4.84%	2.08%	6.16%	7.45%

## Data Availability

The original procedure and dataset for transfer learning are available on request.

## Supplementary Materials

The following supporting information can be downloaded at: https://www.mdpi.com/article/10.3390/s24175726/s1, Supplementary File S1: Codes for image segmentation model based on prior knowledge and deep learning; Supplementary File S2: Codes for root hair thinning, root hair and root axis connecting, root hair separation, and root hair curling angle calculation.

## Abbreviations

X-CT: X-ray computed tomography; MRI: Magnetic resonance imaging; NMR: nuclear magnetic resonance.
