# Supplementary material for: Characterization of Root Hair Curling and Nodule Development in Soybean–Rhizobia Symbiosis
Source: Sensors (Basel). 2024 Sep 3;24(17):5726. doi: 10.3390/s24175726 (PMC11398186; doi:10.3390/s24175726)
Supplement: Supplementary file 1 [file sensors-24-05726-s001.zip › sensors-3023738-supplementary.pdf]

Additional file 1

Code for P-T-U-Net model

```
import numpy as np
import cv2
from PIL import Image
import matplotlib.pyplot as plt
import os
import tensorflow as tf
from keras.models import Model
from keras.layers import Input, concatenate, Conv2D, MaxPooling2D,
UpSampling2D, Reshape, core, Dropout
from keras.optimizers import Adam, SGD
from keras.callbacks import ModelCheckpoint, LearningRateScheduler
from keras import backend as K
config = tf.compat.v1.ConfigProto()
config.gpu_options.allow_growth=True
tf_session =
tf.compat.v1.Session(graph=tf.compat.v1.get_default_graph(),config=config)
tf.compat.v1.keras.backend.set_session(tf_session)

img_x, img_y = (384, 384)
dx = 48
filelst = os.listdir('DRIVE/training/images/')
filelst = ['DRIVE/training/images/' + v for v in filelst]
imgs = [cv2.imread(file) for file in filelst]
filelst = os.listdir('DRIVE/training/1st_manual/')
filelst = ['DRIVE/training/1st_manual/' + v for v in filelst]
manuals = [cv2.imread(file, cv2.IMREAD_GRAYSCALE) for file in filelst]
imgs = [cv2.resize(v, (img_x, img_y)) for v in imgs]
manuals = [cv2.resize(v, (img_x, img_y)) for v in manuals]
X_train = np.array(imgs)
Y_train = np.array(manuals)
X_train = X_train.astype('float32') / 255.
Y_train = Y_train.astype('float32') / 255.
X_train = X_train[..., 1]    # the G channel
X_train = np.array([[X_train[:, v * dx:(v + 1) * dx, vv * dx:(vv + 1) * dx] for v in
range(img_y // dx)] for vv in range(img_x // dx)]).reshape(-1, dx, dx)[:,
np.newaxis, ...]
Y_train = np.array([[Y_train[:, v * dx:(v + 1) * dx, vv * dx:(vv + 1) * dx] for v in
range(img_y // dx)] for vv in range(img_x // dx)]).reshape(-1, dx * dx)[...,
np.newaxis]
  temp = 1 - Y_train
Y_train = np.concatenate([Y_train, temp], axis=2)
log_dir = "logs/"
```

```
def unet_model(n_ch, patch_height, patch_width):
	inputs = Input(shape=(n_ch, patch_height, patch_width))
	conv1 = Conv2D(32, (3, 3), activation='relu', padding='same',
data_format='channels_first')(inputs)
	conv1 = Dropout(0.2)(conv1)
	conv1 = Conv2D(32, (3, 3), activation='relu', padding='same',
data_format='channels_first')(conv1)
	pool1 = MaxPooling2D((2, 2))(conv1)

	conv2 = Conv2D(64, (3, 3), activation='relu', padding='same',
data_format='channels_first')(pool1)
	conv2 = Dropout(0.2)(conv2)
	conv2 = Conv2D(64, (3, 3), activation='relu', padding='same',
data_format='channels_first')(conv2)
	pool2 = MaxPooling2D((2, 2))(conv2)

	conv3 = Conv2D(128, (3, 3), activation='relu', padding='same',
data_format='channels_first')(pool2)
	conv3 = Dropout(0.2)(conv3)
	conv3 = Conv2D(128, (3, 3), activation='relu', padding='same',
data_format='channels_first')(conv3)

	up1 = UpSampling2D(size=(2, 2))(conv3)
	up1 = concatenate([conv2, up1], axis=1)
	conv4 = Conv2D(64, (3, 3), activation='relu', padding='same',
data_format='channels_first')(up1)
	conv4 = Dropout(0.2)(conv4)
	conv4 = Conv2D(64, (3, 3), activation='relu', padding='same',
data_format='channels_first')(conv4)

	up2 = UpSampling2D(size=(2, 2))(conv4)
	up2 = concatenate([conv1, up2], axis=1)
	conv5 = Conv2D(32, (3, 3), activation='relu', padding='same',
data_format='channels_first')(up2)
	conv5 = Dropout(0.2)(conv5)
	conv5 = Conv2D(32, (3, 3), activation='relu', padding='same',
data_format='channels_first')(conv5)

	conv6 = Conv2D(2, (1, 1), activation='relu', padding='same',
data_format='channels_first')(conv5)
	conv6 = core.Reshape((2, patch_height * patch_width))(conv6)
	conv6 = core.Permute((2, 1))(conv6)
```

```
    conv7 = core.Activation('softmax')(conv6)

    model = Model(inputs=inputs, outputs=conv7)
    return model


model = unet_model(X_train.shape[1], X_train.shape[2], X_train.shape[3])
model.summary()

checkpointer = ModelCheckpoint(log_dir + 'ep{epoch:03d}-loss{loss:.3f}-
val_loss{val_loss:.3f}.h5', verbose=1, monitor='val_acc',
                                mode='auto', save_best_only=False, period=2)
model.compile(optimizer=Adam(lr=0.001), loss='categorical_crossentropy',
metrics=['accuracy'])

model.fit(X_train, Y_train, batch_size=64, epochs=128, verbose=2, shuffle=True,
validation_split=0.2,
          callbacks=[checkpointer])
# test
# imgs = cv2.imread('DRIVE/test/images/21.tif')[..., 2]    # the G channel
# imgs = cv2.resize(imgs, (img_x, img_y))
# # manuals = np.asarray(Image.open('DRIVE/test/1st_manual/19.gif'))
# X_test = imgs.astype('float32') / 255.
# # Y_test = manuals.astype('float32') / 255.
# X_test = np.array([[X_test[v * dx:(v + 1) * dx, vv * dx:(vv + 1) * dx] for v in
range(img_y // dx)] for vv in range(img_x // dx)]).reshape(-1, dx, dx)[:,
np.newaxis, ...]
# model.load_weights('best_weights.h5')
# Y_pred = model.predict(X_test)
# Y_pred = Y_pred[..., 0].reshape(img_x // dx, img_y // dx, dx, dx)
# Y_pred = [Y_pred[:, v, ...] for v in range(img_x // dx)]
# Y_pred = np.concatenate(np.concatenate(Y_pred, axis=1), axis=1)
# # Y_pred = cv2.resize(Y_pred, (Y_test.shape[1], Y_test.shape[0]))
# plt.figure(1,figsize=(6, 6))
# plt.imshow(Y_pred)
# plt.show()
# # plt.figure(2,figsize=(6, 6))
# # plt.imshow(Y_test)
# # plt.show()

# predict
import numpy as np
import cv2
from PIL import Image
```

```
import matplotlib.pyplot as plt
import os
from keras.models import Model,load_model
from keras.layers import Input, concatenate, Conv2D, MaxPooling2D,
UpSampling2D, Reshape, core, Dropout
from keras.optimizers import Adam, SGD
from keras.callbacks import ModelCheckpoint, LearningRateScheduler
from keras import backend as K
from keras.models import load_model
from sklearn.metrics import confusion_matrix, f1_score, precision_score,
recall_score, accuracy_score

# load
# model = load_model('./logs/ep128-loss0.141-val_loss0.152.h5')

# initial
img_x, img_y = (384, 384)
dx = 48
i=1
imgs2 = "DRIVE/test/images/"
imgs22 = "DRIVE/test/images_predict/"
# load
model = load_model('./logs/ep128-loss0.116-val_loss0.199.h5')

for aa in os.listdir(imgs2):
    aaa = os.path.join(imgs2, aa)
    imgs = cv2.imread(aaa)[..., 1]
    imgs = cv2.resize(imgs, (img_x, img_y))
    manuals = cv2.imread('./DRIVE/training/images/001.jpg',
cv2.IMREAD_GRAYSCALE)
    manuals = np.array(manuals)
    X_test = imgs.astype('float32') / 255.
    Y_test = manuals.astype('float32') / 255.
    X_test = np.array([[X_test[v * dx:(v + 1) * dx, vv * dx:(vv + 1) * dx] for v in
range(img_y // dx)] for vv in range(img_x // dx)]).reshape(-1, dx, dx)[:,
np.newaxis, ...]
    # predict
    Y_pred = model.predict(X_test)
    Y_pred = Y_pred[..., 0].reshape(img_x // dx, img_y // dx, dx, dx)
    Y_pred = [Y_pred[:, v, ...] for v in range(img_x // dx)]
    Y_pred = np.concatenate(np.concatenate(Y_pred, axis=1), axis=1)
    Y_pred = cv2.resize(Y_pred, (Y_test.shape[1], Y_test.shape[0]))
    # cv2.imwrite(imgs22 + aa[:-4] + '.png', Y_pred)
    i +=1
```

```
    plt.figure(i); plt.imshow(Y_pred, cmap='Greys_r'); plt.savefig(imgs22 + aa[:-4]
+ '.png'); plt.close()
```

Additional file 2

# Root hair thinning

```
import numpy as np
import cv2
import os
import matplotlib.pyplot as plt
from PIL import Image
import imageio

in_path = 'C:/Users/18512/Desktop/20230529/02gray/change4xin-whole.tif'
in_path2 = 'C:/Users/18512/Desktop/20230529/03thinning/'
out_path = 'C:/Users/18512/Desktop/20230529/03thinning/'
image = cv2.imread(in_path)
size = image.shape; m=size[0]; n=size[1]; print(m,n)
image_gray=cv2.cvtColor(image,cv2.COLOR_BGR2GRAY)
ret,image_binary=cv2.threshold(image_gray,127,255,cv2.THRESH_BINARY)
image3=np.zeros((m,n))
for i in range(m):
    for j in range(n):
        if image_binary[i,j] == 0:
            image3[i,j] = 0
        else:
            image3[i,j]=1
# print(image_binary)
print(image3[230,245])
# print(type(image_binary))
def thinning_step1(image):
    image2=np.zeros((m,n))
    for i in range(2,m-1):
        for j in range(2,n-1):
            p1=image[i,j]; p2=image[i-1,j]; p3=image[i-1,j+1]; p4=image[i,j+1];
p5=image[i+1,j+1]
            p6=image[i+1,j]; p7=image[i+1,j-1]; p8=image[i,j-1]; p9=image[i-1,j-1]
            B=p2+p3+p4+p5+p6+p7+p8+p9
            A1=-p2+p3; A2=-p3+p4; A3=-p4+p5; A4=-p5+p6; A5=-p6+p7; A6=-p7+p8; A7=-
p8+p9; A8=-p9+p2
            if (1<B) & (B<7):
                if ((A1==1) & (A2!=1) & (A3!=1) & (A4!=1) & (A5!=1) & (A6!=1) & (A7!=1)
& (A8!=1))|\
                    ((A2==1) & (A1!=1) & (A3!=1) & (A4!=1) & (A5!=1) & (A6!=1) &
```

```
(A7!=1) & (A8!=1))| \
                    ((A3 == 1) & (A1 != 1) & (A2 != 1) & (A4 != 1) & (A5 != 1) & (A6 !=
1) & (A7 != 1) & (A8 != 1)) | \
                    ((A4 == 1) & (A1 != 1) & (A2 != 1) & (A3 != 1) & (A5 != 1) & (A6 !=
1) & (A7 != 1) & (A8 != 1)) | \
                    ((A5 == 1) & (A1 != 1) & (A2 != 1) & (A3 != 1) & (A4 != 1) & (A6 !=
1) & (A7 != 1) & (A8 != 1)) | \
                    ((A6 == 1) & (A1 != 1) & (A2 != 1) & (A3 != 1) & (A4 != 1) & (A5 !=
1) & (A7 != 1) & (A8 != 1)) | \
                    ((A7 == 1) & (A1 != 1) & (A2 != 1) & (A3 != 1) & (A4 != 1) & (A5 !=
1) & (A6 != 1) & (A8 != 1)) | \
                    ((A8 == 1) & (A1 != 1) & (A2 != 1) & (A3 != 1) & (A4 != 1) & (A5 !=
1) & (A6 != 1) & (A7 != 1)) :
                    if p2*p4*p6==0:
                        if p4*p6*p8==0:
                            image2[i,j]=0
                        else:
                            image2[i,j]=image[i,j]
                    else:
                        image2[i,j]=image[i,j]
                else:
                    image2[i,j]=image[i,j]
            else:
                image2[i,j]=image[i,j]
    return image2

def thinning_step2(image):
    image2=np.zeros((m,n))
    for i in range(2,m-1):
        for j in range(2,n-1):
            p1 = image[i, j]; p2 = image[i - 1, j]; p3 = image[i - 1, j + 1]; p4 = image[i, j + 1]; p5
= image[i + 1, j + 1]
            p6 = image[i + 1, j]; p7 = image[i + 1, j - 1]; p8 = image[i, j - 1]; p9 = image[i - 1, j
- 1]
            B=p2+p3+p4+p5+p6+p7+p8+p9
            A1=-p2+p3; A2=-p3+p4; A3=-p4+p5; A4=-p5+p6; A5=-p6+p7; A6=-p7+p8; A7=-
p8+p9; A8=-p9+p2
            if (1<B) & (B<7):
                if ((A1==1) & (A2!=1) & (A3!=1) & (A4!=1) & (A5!=1) & (A6!=1) & (A7!=1)
& (A8!=1))|\
                    ((A2==1) & (A1!=1) & (A3!=1) & (A4!=1) & (A5!=1) & (A6!=1) &
(A7!=1) & (A8!=1))| \
                    ((A3 == 1) & (A1 != 1) & (A2 != 1) & (A4 != 1) & (A5 != 1) & (A6 !=
1) & (A7 != 1) & (A8 != 1)) | \
```

```
                    ((A4 == 1) & (A1 != 1) & (A2 != 1) & (A3 != 1) & (A5 != 1) & (A6 != 
1) & (A7 != 1) & (A8 != 1)) | \
                    ((A5 == 1) & (A1 != 1) & (A2 != 1) & (A3 != 1) & (A4 != 1) & (A6 != 
1) & (A7 != 1) & (A8 != 1)) | \
                    ((A6 == 1) & (A1 != 1) & (A2 != 1) & (A3 != 1) & (A4 != 1) & (A5 != 
1) & (A7 != 1) & (A8 != 1)) | \
                    ((A7 == 1) & (A1 != 1) & (A2 != 1) & (A3 != 1) & (A4 != 1) & (A5 != 
1) & (A6 != 1) & (A8 != 1)) | \
                    ((A8 == 1) & (A1 != 1) & (A2 != 1) & (A3 != 1) & (A4 != 1) & (A5 != 
1) & (A6 != 1) & (A7 != 1)):
                if p2*p4*p8==0:
                    if p2*p6*p8==0:
                        image2[i,j]=0
                    else:
                        image2[i,j]=image[i,j]
                else:
                    image2[i,j]=image[i,j]
            else:
                image2[i,j]=image[i,j]
        else:
            image2[i,j]=image[i,j]
    return image2

def savefigure(data):
        data = (data * 255.0).astype('uint8')
    plt.figure(figsize=(3.84, 3.84), dpi=100)
    new_im = Image.fromarray(data, mode='L')
    plt.imshow(new_im)
    plt.axis('off')
    plt.gca().xaxis.set_major_locator(plt.NullLocator())
    plt.gca().yaxis.set_major_locator(plt.NullLocator())
    plt.subplots_adjust(top=1, bottom=0, right=1, left=0, hspace=0, wspace=0)
    plt.margins(0, 0)
    imageio.imsave(out_path + in_path[39:-4] + '.png', new_im)

i = 1
image33=image3
while i<20:
    image1 = thinning_step1(image33)
    image2 = thinning_step2(image1)
    image33=image2
    i=i+1

savefigure(image2)
```

```
# plt.imshow(image2, cmap='Greys_r'); plt.savefig(in_path2 + in_path[39:-4] + '.png'); plt.close()
```

# Remove root axis

```
import numpy as np
import cv2
import os
import matplotlib.pyplot as plt
from PIL import Image
import imageio

in_path = 'C:/Users/18512/Desktop/20230529/03thinning/change4xin-whole.png'
in_path2 = 'C:/Users/18512/Desktop/20230529/02gray/change4xin-core.tif'
out_path = 'C:/Users/18512/Desktop/20230529/04thinning-without-core/'

image = cv2.imread(in_path); image22 = cv2.imread(in_path2)
size = image.shape; m=size[0]; n=size[1]; print(m,n)
image_gray=cv2.cvtColor(image,cv2.COLOR_BGR2GRAY);
image_gray22=cv2.cvtColor(image22,cv2.COLOR_BGR2GRAY)
ret,image_binary=cv2.threshold(image_gray,127,255,cv2.THRESH_BINARY);
ret,image_binary22=cv2.threshold(image_gray22,127,255,cv2.THRESH_BINARY)
image3=np.zeros((m,n)); image33 = np.zeros((m,n)); image44 = np.zeros((m,n))
for i in range(m):
    for j in range(n):
        if image_binary[i,j] == 0:
            image3[i,j] = 0
        else:
            image3[i,j]=1
print(image3[230,245])

for i in range(m):
    for j in range(n):
        if image_binary22[i,j] == 0:
            image33[i,j] = 0
        else:
            image33[i,j]=1
print(image33[230,245])

for i in range(m):
    for j in range(n):
        if image3[i,j] == 0:
            pass
```

```
        else:
            if image33[i,j] == 0:
                image44[i,j] = image3[i,j]
            else:
                pass
print(image44.sum())

def savefigure(data):
    data = (data * 255.0).astype('uint8')
    plt.figure(figsize=(3.84, 3.84), dpi=100)
    new_im = Image.fromarray(data, mode='L')
    plt.imshow(new_im)
    plt.axis('off')
    plt.gca().xaxis.set_major_locator(plt.NullLocator())
    plt.gca().yaxis.set_major_locator(plt.NullLocator())
    plt.subplots_adjust(top=1, bottom=0, right=1, left=0, hspace=0, wspace=0)
    plt.margins(0, 0)
    imageio.imsave(out_path + in_path[-20:-4] + '.png', new_im)

savefigure(image44)

# plt.imshow(image2, cmap='Greys_r'); plt.savefig(in_path2 + in_path[39:-4] + '.png'); plt.close()
```

# Root axis boundary segmentation

```
import numpy as np
import cv2
import os
import matplotlib.pyplot as plt
from PIL import Image
import imageio

in_path = 'C:/Users/18512/Desktop/20230529/02gray/change4xin-core.tif'
out_path = 'C:/Users/18512/Desktop/20230529/05edgesegmentation/'
image = cv2.imread(in_path)
size = image.shape; m=size[0]; n=size[1]; print('行与列：',m,n)
image_gray=cv2.cvtColor(image,cv2.COLOR_BGR2GRAY)
ret,image_binary=cv2.threshold(image_gray,127,255,cv2.THRESH_BINARY)
image3=np.zeros((m,n)); image4=np.zeros((m,n)); image5=np.zeros((m,n));
image6=np.zeros((m,n)); image7=np.zeros((m,n))
```

```
for i in range(m):
    for j in range(n):
        if image_binary[i,j] == 0:
            image3[i,j] = 0
        else:
            image3[i,j]=1
print(image3.sum())

def neighbor8(image,i,j):
    p1 = image[i, j];                p2 = image[i - 1, j];                p3 = image[i - 1, j + 1]
    p4 = image[i, j + 1];                p5 = image[i + 1, j + 1];                p6 = image[i + 1, j]
    p7 = image[i + 1, j - 1];                p8 = image[i, j - 1];                p9 = image[i - 1, j - 1]
    p10 = p2+p3+p4+p5+p6+p7+p8+p9
    return p1,p2,p3,p4,p5,p6,p7,p8,p9,p10

image = image3
for i in range(2,m-1):
    for j in range(2,n-1):
        if image[i,j] == 1:
            p1, p2, p3, p4, p5, p6, p7, p8, p9, p10 = neighbor8(image, i, j)
            if p10 == 0:
                image3[i,j] = 0
print('sum：',image3.sum())

def edge1(image3):
    image2=np.zeros((m,n))
    for i in range(2,m-1):
        for j in range(2,n-1):
            p1, p2, p3, p4, p5, p6, p7, p8, p9, p10 = neighbor8(image3, i, j)
            B=p2+p3+p4+p5+p6+p7+p8+p9
            A1=-p2+p3; A2=-p3+p4; A3=-p4+p5; A4=-p5+p6; A5=-p6+p7; A6=-p7+p8; A7=-
p8+p9; A8=-p9+p2
            if (1<B) & (B<7):
                if ((A1==1) & (A2!=1) & (A3!=1) & (A4!=1) & (A5!=1) & (A6!=1) & (A7!=1)
& (A8!=1))|\
                    ((A2==1) & (A1!=1) & (A3!=1) & (A4!=1) & (A5!=1) & (A6!=1) &
(A7!=1) & (A8!=1))| \
                        ((A3 == 1) & (A1 != 1) & (A2 != 1) & (A4 != 1) & (A5 != 1) & (A6 !=
1) & (A7 != 1) & (A8 != 1)) | \
                        ((A4 == 1) & (A1 != 1) & (A2 != 1) & (A3 != 1) & (A5 != 1) & (A6 !=
1) & (A7 != 1) & (A8 != 1)) | \
                        ((A5 == 1) & (A1 != 1) & (A2 != 1) & (A3 != 1) & (A4 != 1) & (A6 !=
1) & (A7 != 1) & (A8 != 1)) | \
                        ((A6 == 1) & (A1 != 1) & (A2 != 1) & (A3 != 1) & (A4 != 1) & (A5 !=
```

```
1) & (A7 != 1) & (A8 != 1)) | \
                              ((A7 == 1) & (A1 != 1) & (A2 != 1) & (A3 != 1) & (A4 != 1) & (A5 !=
1) & (A6 != 1) & (A8 != 1)) | \
                              ((A8 == 1) & (A1 != 1) & (A2 != 1) & (A3 != 1) & (A4 != 1) & (A5 !=
1) & (A6 != 1) & (A7 != 1)) :
                          if p2*p4*p6==0:
                              if p4*p6*p8==0:
                                  image2[i,j]=image3[i,j]
                              else:
                                  pass
                          else:
                              pass
                      else:
                          pass
                  else:
                      pass
    print('sum boundary1：', image2.sum())
    return image2
image4 = edge1(image3)

def edge2(image3):
    image2 = np.zeros((m, n))
    for i in range(2,m-1):
        for j in range(2,n-1):
            p1, p2, p3, p4, p5, p6, p7, p8, p9, p10 = neighbor8(image3, i, j)
            B = p2 + p3 + p4 + p5 + p6 + p7 + p8 + p9
            A1 = -p2 + p3;          A2 = -p3 + p4;          A3 = -p4 + p5
            A4 = -p5 + p6;          A5 = -p6 + p7;          A6 = -p7 + p8
            A7 = -p8 + p9;          A8 = -p9 + p2
            if p1 == 0:
                pass
            else:
                if p10 < 7:
                    if ((A1 == 1) & (A2 != 1) & (A3 != 1) & (A4 != 1) & (A5 != 1) & (A6 !=
1) & (A7 != 1) & (A8 != 1)) | \
                              ((A2 == 1) & (A1 != 1) & (A3 != 1) & (A4 != 1) & (A5 != 1) &
(A6 != 1) & (A7 != 1) & (
                                      A8 != 1)) | \
                              ((A3 == 1) & (A1 != 1) & (A2 != 1) & (A4 != 1) & (A5 != 1) &
(A6 != 1) & (A7 != 1) & (
                                      A8 != 1)) | \
                              ((A4 == 1) & (A1 != 1) & (A2 != 1) & (A3 != 1) & (A5 != 1) &
(A6 != 1) & (A7 != 1) & (
                                      A8 != 1)) | \
```

```
                                        ((A5 == 1) & (A1 != 1) & (A2 != 1) & (A3 != 1) & (A4 != 1) &
(A6 != 1) & (A7 != 1) & (
                                                A8 != 1)) | \
                                        ((A6 == 1) & (A1 != 1) & (A2 != 1) & (A3 != 1) & (A4 != 1) &
(A5 != 1) & (A7 != 1) & (
                                                A8 != 1)) | \
                                        ((A7 == 1) & (A1 != 1) & (A2 != 1) & (A3 != 1) & (A4 != 1) &
(A5 != 1) & (A6 != 1) & (
                                                A8 != 1)) | \
                                        ((A8 == 1) & (A1 != 1) & (A2 != 1) & (A3 != 1) & (A4 != 1) &
(A5 != 1) & (A6 != 1) & (A7 != 1)):
                                    if p2 * p4 * p8 == 0:
                                        if p2 * p6 * p8 == 0:
                                            image2[i, j] = image3[i,j]
                                        else:
                                            pass
                                    else:
                                        pass
                                else:
                                    pass
    print(' um boundary2：', image2.sum())
    return image2
image5 = edge2(image3)

def edge3(image3):
    image2 = np.zeros((m, n))
    for i in range(2, m - 1):
        for j in range(2, n - 1):
            p1, p2, p3, p4, p5, p6, p7, p8, p9, p10 = neighbor8(image3, i, j)
            if p1 == 0:
                pass
            else:
                if p10 < 6:
                    image2[i,j] = image3[i,j]
                else:
                    pass
    print(' um boundary3：', image2.sum())
    return image2
image6 = edge3(image3)

for i in range(m):
    for j in range(n):
        if (image4[i,j]) or (image5[i,j]) or (image6[i,j]):
            image7[i,j] = 1
```

```
        else:
            pass

def savefigure(data):
    data = (data * 255.0).astype('uint8')
    plt.figure(figsize=(3.84, 3.84), dpi=100)
    new_im = Image.fromarray(data, mode='L')
    plt.imshow(new_im)
    plt.axis('off')
    plt.gca().xaxis.set_major_locator(plt.NullLocator())
    plt.gca().yaxis.set_major_locator(plt.NullLocator())
    plt.subplots_adjust(top=1, bottom=0, right=1, left=0, hspace=0, wspace=0)
    plt.margins(0, 0)
    imageio.imsave(out_path + in_path[-20:-4] + '.png', new_im)

savefigure(image7)
```

# Root axis and root hair connection

```
import numpy as np
import cv2
import os
import matplotlib.pyplot as plt
from PIL import Image
import imageio

in_path = 'C:/Users/18512/Desktop/20230529/04thinning-without-core/change4xin-whole.png'
in_path2 = 'C:/Users/18512/Desktop/20230529/05edgesegmentation/change4xin-core.png'
out_path = 'C:/Users/18512/Desktop/20230529/06thinning-with-core-border/'
image = cv2.imread(in_path); image22 = cv2.imread(in_path2)
size = image.shape; m=size[0]; n=size[1]; print(m,n)
image_gray=cv2.cvtColor(image,cv2.COLOR_BGR2GRAY);
image_gray22=cv2.cvtColor(image22,cv2.COLOR_BGR2GRAY)
ret,image_binary=cv2.threshold(image_gray,127,255,cv2.THRESH_BINARY);
ret,image_binary22=cv2.threshold(image_gray22,127,255,cv2.THRESH_BINARY)
image3=np.zeros((m,n)); image33 = np.zeros((m,n)); image44 = np.zeros((m,n))
for i in range(m):
    for j in range(n):
        if image_binary[i,j] == 0:
            image3[i,j] = 0
        else:
```

```
            image3[i,j]=1
print(image3.sum())

for i in range(m):
    for j in range(n):
        if image_binary22[i,j] == 0:
            image33[i,j] = 0
        else:
            image33[i,j]=1
print(image33[230,245])

#
# image = image3
# for i in range(2,m-1):
#     for j in range(2,n-1):
#         if image[i,j] == 1:
#             p1, p2, p3, p4, p5, p6, p7, p8, p9, p10 = neighbor8(image, i, j)
#             if p10 == 0:
#                 image3[i,j] = 0
# print('sum：',image3.sum())

for i in range(m):
    for j in range(n):
        if (image3[i,j]==1) or (image33[i,j]==1):
            image44[i,j] = 1
        else:
            pass

def savefigure(data):
        data = (data * 255.0).astype('uint8')
    plt.figure(figsize=(3.84, 3.84), dpi=100)
    new_im = Image.fromarray(data, mode='L')
    plt.imshow(new_im)
    plt.axis('off')
    plt.gca().xaxis.set_major_locator(plt.NullLocator())
    plt.gca().yaxis.set_major_locator(plt.NullLocator())
    plt.subplots_adjust(top=1, bottom=0, right=1, left=0, hspace=0, wspace=0)
    plt.margins(0, 0)
    imageio.imsave(out_path + in_path[-20:-10] + '.png', new_im)

savefigure(image44)
```

# Root hair separation

```
import numpy as np
import cv2
import os
import matplotlib.pyplot as plt
from PIL import Image
import imageio

in_path = 'C:/Users/18512/Desktop/20230529/04thinning-without-core/temple4-whole.png'
out_path = 'C:/Users/18512/Desktop/20230529/temple4-hairs/'
image = cv2.imread(in_path)
size = image.shape; m=size[0]; n=size[1]; print('row and column：',m,n)
image_gray=cv2.cvtColor(image,cv2.COLOR_BGR2GRAY)
ret,image_binary=cv2.threshold(image_gray,127,255,cv2.THRESH_BINARY)
image3=np.zeros((m,n));          image4=np.zeros((m,n));          image5=np.zeros((m,n));
image6=np.zeros((m,n))
    for j in range(n):
        if image_binary[i,j] == 0:
            image3[i,j] = 0
        else:
            image3[i,j]=1
print(image3.sum())

def neighbor8(image,i,j):
    p1 = image[i, j];        p2 = image[i - 1, j];        p3 = image[i - 1, j + 1]
    p4 = image[i, j + 1];        p5 = image[i + 1, j + 1];        p6 = image[i + 1, j]
    p7 = image[i + 1, j - 1];        p8 = image[i, j - 1];        p9 = image[i - 1, j - 1]
    p10 = p2+p3+p4+p5+p6+p7+p8+p9
    return p1,p2,p3,p4,p5,p6,p7,p8,p9,p10

image = image3
for i in range(2,m-1):
    for j in range(2,n-1):
        if image[i,j] == 1:
            p1, p2, p3, p4, p5, p6, p7, p8, p9, p10 = neighbor8(image, i, j)
            if p10 == 0:
                image3[i,j] = 0
print('sum：',image3.sum())

def locatingP1(data):
    for i in range(2,m-1):
```

```
        for j in range(2,n-1):
            p1, p2, p3, p4, p5, p6, p7, p8, p9, p10 = neighbor8(data, i, j)
            if (p1==1) & (p10==1):
                return i,j
                break
            else:
                pass
x,y = locatingP1(image3); print('x,y:',x,y)

xx = []; yy = []
for i in range(2, m - 1):
    for j in range(2, n - 1):
        p1, p2, p3, p4, p5, p6, p7, p8, p9, p10 = neighbor8(image3, i, j)
        if (p1 == 1) & (p10 == 1):
            xx.append(i); yy.append(j)
        else:
            pass
print('abscissa',xx); print('ordinate',yy)
# for i in range(len(xx)):
#       image5[xx[i],yy[i]] = image3[xx[i],yy[i]]

def finding_link(x,y,image3):
    image4[x,y] = image3[x,y]; image = image3 - image4
    p1, p2, p3, p4, p5, p6, p7, p8, p9, p10 = neighbor8(image, x, y)
    ZuoBZ = [p2,p3,p4,p5, p6, p7, p8, p9]
    HengZB = [x-1,x-1,x,x+1,x+1,x+1,x,x-1]
    ZongZB = [y,y+1,y+1,y+1,y,y-1,y-1,y-1]
    if p10 == 1:
        for i in range(len(ZuoBZ)):
            if ZuoBZ[i] == 0:
                continue
            else:
                x = HengZB[i]; y = ZongZB[i]; finding_link(x,y,image3)
    else:
        pass
    return image4

for i in range(len(xx)):
    x = xx[i]; y = yy[i]; print('###################abscissa 、
ordinate#######################:',x,y)
    image5 = finding_link(x, y, image3); print('root hair length/true pixel', image5.sum())
    data = (image5 * 255.0).astype('uint8')
    plt.figure(figsize=(3.84, 3.84), dpi=100)
    new_im = Image.fromarray(data, mode='L')
```

```
    plt.imshow(new_im)

    plt.axis('off')
    plt.gca().xaxis.set_major_locator(plt.NullLocator())
    plt.gca().yaxis.set_major_locator(plt.NullLocator())
    plt.subplots_adjust(top=1, bottom=0, right=1, left=0, hspace=0, wspace=0)
    plt.margins(0, 0)

    imageio.imsave(out_path + in_path[-17:-4] + str(i) + '.png', new_im)
```

```
import numpy as np
import cv2
import os
import matplotlib.pyplot as plt
from PIL import Image
import imageio
in_path = 'C:/Users/18512/Desktop/20230529/temple2-hairs/'
out_path = 'C:/Users/18512/Desktop/20230529/temple2-hairs - 2/'
i = 0; name = []
for img_name in os.listdir(in_path):
    in_path_name = os.path.join(in_path, img_name)
    name.append(in_path_name)
ShuL = len(name); MingC = len(name[0]); print('image number，name length：',ShuL,MingC)
name_2 = name[0]; print(name_2)
name_3 = name_2[0:-5]; print(name_3)

# def Numpic(data_name):
#     image = cv2.imread(data_name); size = image.shape; m = size[0]; n = size[1]; image3 = np.zeros((m,n))
#     image_gray = cv2.cvtColor(image, cv2.COLOR_BGR2GRAY);   ret, image_binary = cv2.threshold(image_gray, 127, 255, cv2.THRESH_BINARY)
#     for i in range(m):
#         for j in range(n):
#             if image_binary[i, j] == 0:
#                 image3[i, j] = 0
#             else:
#                 image3[i, j] = 1
#     return image3

def Savepic(image,i):
    data = (image * 255.0).astype('uint8')
    plt.figure(figsize=(3.84, 3.84), dpi=100)
```

```
        new_im = Image.fromarray(data, mode='L')
        plt.imshow(new_im)
        plt.axis('off')
        plt.gca().xaxis.set_major_locator(plt.NullLocator())
        plt.gca().yaxis.set_major_locator(plt.NullLocator())
        plt.subplots_adjust(top=1, bottom=0, right=1, left=0, hspace=0, wspace=0)
        plt.margins(0, 0)
        imageio.imsave(out_path + name_2[-18:-5] + str(i) + '.png', new_im)

for ii in range(ShuL-1):
    data_name = name_3 + str(ii) + '.png'; ii_2 = ii + 1; data_name2 = name_3 + str(ii_2) + '.png'
    image = cv2.imread(data_name); size = image.shape; m = size[0]; n = size[1]
    image_gray = cv2.cvtColor(image, cv2.COLOR_BGR2GRAY); ret, image_binary = cv2.threshold(image_gray, 127, 255, cv2.THRESH_BINARY)
    image3 = np.zeros((m, n)); image4 = np.zeros((m, n)); image5 = np.zeros((m, n)); image6 = np.zeros((m, n))
    for i in range(m):
        for j in range(n):
            if image_binary[i, j] == 0:
                image3[i, j] = 0
            else:
                image3[i, j] = 1

    image2 = cv2.imread(data_name2); size = image2.shape; m = size[0]; n = size[1]
    image_gray2 = cv2.cvtColor(image2, cv2.COLOR_BGR2GRAY); ret2, image_binary2 = cv2.threshold(image_gray2, 127, 255, cv2.THRESH_BINARY)
    for i in range(m):
        for j in range(n):
            if image_binary2[i, j] == 0:
                image4[i, j] = 0
            else:
                image4[i, j] = 1

    if ii == 0:
        image5 = image3
        Savepic(image5,ii)
    else:
        image5 = image4 - image3
        Savepic(image5,ii)


# Curling angle calculation
import numpy as np
import cv2
```

```
import os
import matplotlib.pyplot as plt
from PIL import Image

in_path = 'C:/Users/18512/Desktop/20230529/temple4-hairs -2/'
out_path = 'C:/Users/18512/Desktop/20230529/temple1-hairs -2/'
i = 0; name = []; Hengzb2 = []; Zongzb2 = []; Jiaod2 = []

def neighbor8(image,i,j):
    p1 = image[i, j];        p2 = image[i - 1, j];        p3 = image[i - 1, j + 1]
    p4 = image[i, j + 1];        p5 = image[i + 1, j + 1];        p6 = image[i + 1, j]
    p7 = image[i + 1, j - 1];        p8 = image[i, j - 1];        p9 = image[i - 1, j - 1]
    p10 = p2+p3+p4+p5+p6+p7+p8+p9
    return p1,p2,p3,p4,p5,p6,p7,p8,p9,p10
def locatingP1(data):
    for i in range(2,m-1):
        for j in range(2,n-1):
            p1, p2, p3, p4, p5, p6, p7, p8, p9, p10 = neighbor8(data, i, j)
            if (p1==1) & (p10==1):
                return i,j
                break
            else:
                pass
def finding_link(x,y,image3):
    image4[x,y] = image3[x,y]; image = image3 - image4
    p1, p2, p3, p4, p5, p6, p7, p8, p9, p10 = neighbor8(image, x, y)
    ZuoBZ = [p2,p3,p4,p5, p6, p7, p8, p9]
    HengZB = [x-1,x-1,x,x+1,x+1,x+1,x,x-1]; Hengzb2.append(x)
    ZongZB = [y,y+1,y+1,y+1,y,y-1,y-1,y-1]; Zongzb2.append(y)
    if p10 == 1:
        for i in range(len(ZuoBZ)):
            if ZuoBZ[i] == 0:
                continue
            else:
                x = HengZB[i]; y = ZongZB[i]; finding_link(x,y,image3)
    else:
        pass
    return Hengzb2,Zongzb2

for img_name in os.listdir(in_path):
    in_path_name = os.path.join(in_path, img_name)
    name.append(in_path_name)
    image = cv2.imread(in_path_name); size = image.shape; m = size[0]; n = size[1]
    image_gray = cv2.cvtColor(image, cv2.COLOR_BGR2GRAY); ret, image_binary =
```

```
cv2.threshold(image_gray, 127, 255, cv2.THRESH_BINARY)
    image3 = np.zeros((m, n)); image4 = np.zeros((m, n)); image5 = np.zeros((m, n)); image6 =
np.zeros((m, n))
    for i in range(m):
        for j in range(n):
            if image_binary[i, j] == 0:
                image3[i, j] = 0
            else:
                image3[i, j] = 1
    if ((image3.sum()) > 6):
        xx=[]; yy=[]
        for i in range(2, m - 1):
            for j in range(2, n - 1):
                p1, p2, p3, p4, p5, p6, p7, p8, p9, p10 = neighbor8(image3, i, j)
                if (p1 == 1) & (p10 == 1):
                    xx,yy = finding_link(i,j,image3)
                else:
                    pass
        xx_len = len(xx)
        x1 = xx[0]; x2 = xx[1]; x3 = xx[2]; x4 = xx[xx_len-3]; x5 = xx[xx_len-2]; x6 = xx[xx_len-
1]
        y1 = yy[0]; y2 = yy[1]; y3 = yy[2]; y4 = yy[xx_len-3]; y5 = yy[xx_len-2]; y6 = yy[xx_len-
1]
        JiaoD_m = (x3 - x1) * (x6 - x4) + (y3 - y1) * (y6 -y4)
        JiaoD_z1 = np.sqrt((x3-x1)^2 + (y3-y1)^2); JiaoD_z2 = np.sqrt((x6-x4)^2 + (y6-y4)^2);
JiaoD_z = JiaoD_z1*JiaoD_z2
        JiaoD = JiaoD_m / JiaoD_z
        Jiaod2.append(JiaoD)

    else:
        pass
print(Jiaod2)
angle = 0; j = 0
for i in range(len(Jiaod2)):
    if (np.abs(Jiaod2[i]) > 10):
        pass
    else:
        if not np.isnan(Jiaod2[i]):
            angle = angle + np.abs(Jiaod2[i])
            j = j + 1
        else:
            pass
angle2 = angle / j
print(angle); print('**'); print(j); print('**'); print(angle2)
```

```
#   print(xx);   print('**********************');   print(yy);   print('**********************'); print(JiaoD)
#  print(x1,x2,x3,x4,x5,x6);  print('**');  print(y1,y2,y3,y4,y5,y6);  print('****');  print(JiaoD_m); print(('**')); print(JiaoD_z)
# ShuL = len(name); MingC = len(name[0]); print('image number，  name length：  ',ShuL,MingC)
# name_2 = name[0]; print(name_2)
# name_3 = name_2[0:-5]; print(name_3)
```
